# A wireless endoscopy capsule suitable for imaging of the equine stomach and small intestine

**DOI:** 10.1111/jvim.15825

**Published:** 2020-06-08

**Authors:** Mei Steinmann, Rebecca J. Bezugley, Stephanie L. Bond, Jill S. Pomrantz, Renaud Léguillette

**Affiliations:** ^1^ Faculty of Veterinary Medicine, Department of Veterinary Clinical and Diagnostic Services University of Calgary Calgary Alberta Canada; ^2^ Moore Equine Veterinary Centre Rocky View County Alberta Canada; ^3^ Infiniti Medical, LLC Menlo Park California USA

**Keywords:** capsule endoscopy, endoscopy, gastroscopy

## Abstract

**Background:**

Capsule endoscopy offers a new method for visualization of the gastrointestinal mucosa in horses where other imaging technologies have diagnostic limitations.

**Objectives:**

To (1) test the feasibility of using this novel endoscopy capsule to visualize intestinal mucosa in horses, including an objective assessment of image quality, (2) assess how changes in preadministration preparation affect the transit time and the amount of gastrointestinal mucosa visualized, and (3) describe intestinal mucosa lesions in healthy horses.

**Animals:**

Five healthy adult horses.

**Methods:**

Three protocols were used in a crossover study design. Protocols varied in time fasted, amount of oral fluid administered, and exercise. Manure was radiographically inspected for capsule recovery. Percentage of visible gastrointestinal mucosa was objectively assessed.

**Results:**

Detailed images of the gastrointestinal mucosa were recorded with all 3 protocols, including images of the pylorus, major duodenal papilla, individual villi, and ileocecal junction. Visualization of large intestinal mucosa was poor. Interobserver agreement on image quality was excellent. Capsule administration after feed withholding for 24 hours provided the greatest percentage of visible mucosa in the stomach and small intestine. Total transit time to capsule excretion was 6.5 (3‐8.75) days. Of 15 capsules administered, 3 were not recovered. Lesions visualized included mucosal erosion, ulceration and hemorrhage, areas of thickened mucosa, and evidence of parasitism.

**Conclusions:**

This novel endoscopic capsule appears safe, practical, and noninvasive in horses; however, variability in capsule excretion time must be taken into account for clinical application.

## 1 INTRODUCTION

It can be frustrating for clinicians to diagnose intestinal lesions in horses because no methods allow a thorough examination of the intestinal tract from an internal perspective aboral to the stomach. Imaging techniques are mainly limited to gastric and pyloric examination using long endoscopes, or to ultrasonography of the abdomen, which allows visualization of only a portion of the intestinal tract and rarely allows visualization of intraluminal structures. Capsule endoscopy offers a new method for the visualization of gastrointestinal mucosa in horses where traditional endoscopy has technological and diagnostic limitations.^1^ Approved for human use for over a decade,^2^ capsule endoscopy is superior to other diagnostic methods in detecting small bowel objects, locating sources of gastrointestinal bleeding, and evaluating Crohn's disease while being less invasive.^3^, ^4^ Capsule endoscopy has been used successfully in dogs to evaluate both small and large intestinal mucosa.^5^, ^6^, ^7^

Several capsule systems have been used in horses and ponies to measure gastric emptying time, and to visualize mucosal shape, color, and villus structure in the duodenum and jejunum.^8^, ^9^ These capsule systems travel through the gastrointestinal tract by the action of peristalsis and use radiofrequency technology coupled with external sensors to receive data from the capsule. However, this can be problematic in adult horses with larger body mass because of decrease in signal strength, which can result in intermittent loss of communication between the capsule signal and the external sensors.^9^ Furthermore, the diagnostic capability of these capsule systems is further impeded by short battery life and limited viewing angles.^8^, ^10^, ^11^ Recently, another endoscopic capsule was tested in horses that uses electric‐field propagation to communicate with external sensors. While it has a battery life of approximately 12 hours, image transmission remained inconsistent and diagnostic imaging time was short (0.5‐82.41 minutes).^12^

Recently, a novel wireless endoscopy capsule (also called ambulatory light‐based imaging: ALICAM, Infiniti Medical, Redwood City, California) has been used in dogs to visualize the mucosa of the gastrointestinal tract including the colon, and could detect intraluminal erosions and ulcers, intestinal parasites, motility disorders, inflammatory mucosal changes, and indications of neoplasia.^13^, ^14^ Based on the visible light spectrum (*λ* = 390‐700 nm), this capsule uses 4 cameras and an internal LED light source to capture high resolution, 360° diagnostic images of the gastrointestinal tract. This technology allows animals to be completely ambulatory and engage in normal daily activities as images are stored in an onboard memory system, eliminating the need for external sensors. Furthermore, the capsule also contains an accelerometer that allows it to enter a power‐saving mode when the capsule is not moving, thus maximizing battery life and total imaging time. This technology might have practical applications in equine medicine, but its safety, assessment of image quality, and administration protocols have not yet been established.

Our hypothesis was that the ALICAM would enable safe intraluminal visualization of the gastrointestinal tract mucosa. The objectives of our study were therefore: (1) to test the feasibility of using ALICAM to visualize intestinal mucosa in horses, including an objective assessment of image quality, (2) to assess how changes in preadministration preparation affect both the transit time and the amount of gastrointestinal mucosa visualized, and (3) to describe intestinal mucosal lesions in clinically healthy horses.

## 2 MATERIALS AND METHODS

### 2.1 Animals

Five adult horses from the University of Calgary's teaching herd, including 3 mares and 2 geldings (2 Thoroughbreds, 1 Standardbred, 1 Quarter Horse, 1 Paint), were studied. The horses' health had been followed for greater than 6 months; criteria for inclusion in our study were no abnormalities detected on clinical examination and abdominal ultrasonographic examination. Horses had no previous history of gastrointestinal disease. Horses had a median age of 12 years (interquartile range (IQR) = 5‐15 years), with a median weight of 450 kg (IQR = 456‐546 kg). Horses were maintained on a hay‐only diet throughout the study period.

### 2.2 Study design

Three different protocols were used in a randomized crossover study design with a 2‐week washout period. Protocols differed in terms of exercise, duration off feed, and water intake before capsule administration. In all protocols, capsules were administered on day 0, with manure collection commencing on day 1.

#### 2.2.1 Protocol 1: Low exercise with controlled hay and water intake

Horses were acclimated into stalls and had all feed withdrawn 24 hours before capsule administration. During the initial feed with‐holding period, horses were hand walked twice a day, for approximately 5 to 10 minutes each walk. For the remainder of protocol 1, horses were not exercised. Water access was removed 12 hours before capsule administration. A nasogastric tube was used to facilitate capsule insertion on the morning of day 0, with 0.5 to 1 L of water being administered to aid passage. Water access was replaced 3 hours after capsule administration, and horses were started on a slow refeeding schedule 12 hours after capsule administration. Horses were then fed 4 times per day with a hay‐only diet.

#### 2.2.2 Protocol 2: Increased exercise

The design of protocol 2 was similar to protocol 1, with the addition of 4 hand walks on the day before capsule administration, 4 hand walks on the day of capsule administration (day 0), then 2 hand walks on each day thereafter (day 1 onward). All hand walks were between 5 and 10 minutes in duration.

#### 2.2.3 Protocol 3: Increased feed withholding period

The feed withholding period was increased to 48 hours before capsule administration. On the first day of feed withholding (day −2), a nasogastric tube was used to deliver 8.5 L of water, then 10 L of an electrolyte solution (53 g NaCl, 36 g KCl, 52 g NaHCO_3_ per 10 L) was given twice on day −1. As in protocol 2, horses were hand walked 4 times on day −1, 4 times on day 0, and 4 times on day 1. Horses were then hand walked twice on each following day (day 2 and onward). Two horses were kept in outdoor pens due to behavioral reasons, while 3 horses were kept in stalls.

### 2.3 Sedation

To facilitate nasogastric tubing and administration of the capsule and fluids, xylazine (100 mg/mL), acepromazine (25 mg/mL), or a combination of the 2 were used to effect in 9 of the 15 trials.

### 2.4 Endoscopy capsule technology

The ALICAM system comprises a 11 mm diameter × 33 mm length capsule, containing 4 microcameras installed at 90° angles within the capsule to generate full frames of 4 images acquired simultaneously. These images are acquired at a rate of 20 images/s when the camera is activated by the motion sensors (Figure 3) and stored on an internal memory chip within the capsule's circuitry. After capsule collection upon excretion, the frames were retrieved using a specific reading hardware (ALICAM reader, Infinity Medical, Redwood City, California) and each frame consisted of 4 images side‐by‐side covering a 360° view by the 4 cameras (Figure 3). All the frames were numbered during recording and transferred on a cloud data storage system before analysis. The frames were downloaded and reviewed using an imaging software (ALICAM software, Infinity Medical) that allowed playback on a frame‐by‐frame basis, annotating and time‐tracking of the images.

### 2.5 Endoscopy capsule collection

Manure collection began the day after capsule administration (day 1). Some horses (9/15 data points) wore a manure collection bag (Catch It! Manure Bag, Working Horse Tack, Millersburg, Ohio) and stalls were cleaned normally for others. Manure was collected into separate 64 L plastic bins labeled for each day, and was visually inspected for the capsule during collection; all bins were radiographed if the capsule was not seen with the naked eye. Protocols were followed until the capsule was recovered or for 14 days, at which point all stalled horses were moved back into outdoor pens and the study protocol was ended, per the animal care protocol requirements. Manure collection continued until at least day 24 through cleaning the pens if the capsule was not retrieved before then.

### 2.6 Capsule transit time

**FIGURE 1:**
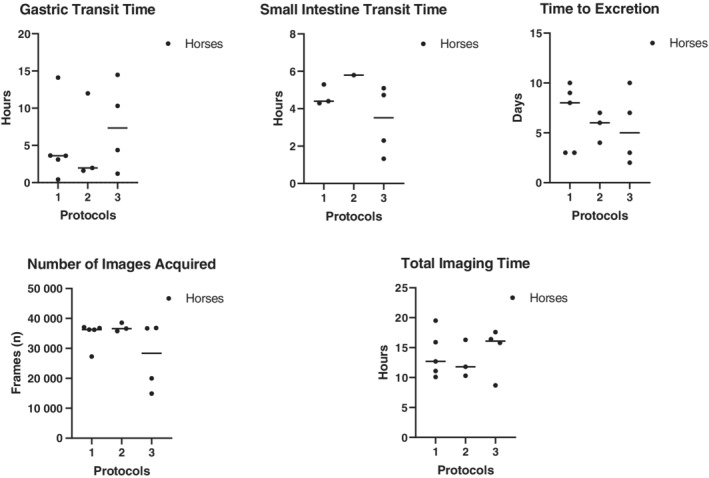
Transit times of capsule through the gastrointestinal tract, total imaging time, and total number of images taken per horse for all protocols. Capsules were not recovered (N/R) in 2 horses in protocol 2 and 1 horse in protocol 3

Transit times of the capsule through the stomach and small intestine as well as time spent in the cecum (before the battery died) were recorded. Total imaging time, the number of images captured, and the number of days taken for the capsule to be excreted were also recorded (Figure 1).

### 2.7 Evaluation of capsule image data: Percentage of mucosa visualized

Using the images database obtained as described above, a random selection of 30 full frames (comprised 4 images each, 1 from each camera) of the stomach, 60 full frames of the small intestine, and 30 full frames of the cecum was generated using a random frame number generation function in a database software (Microsoft Excel version 15.24) for each horse for each trial.

**FIGURE 2:**
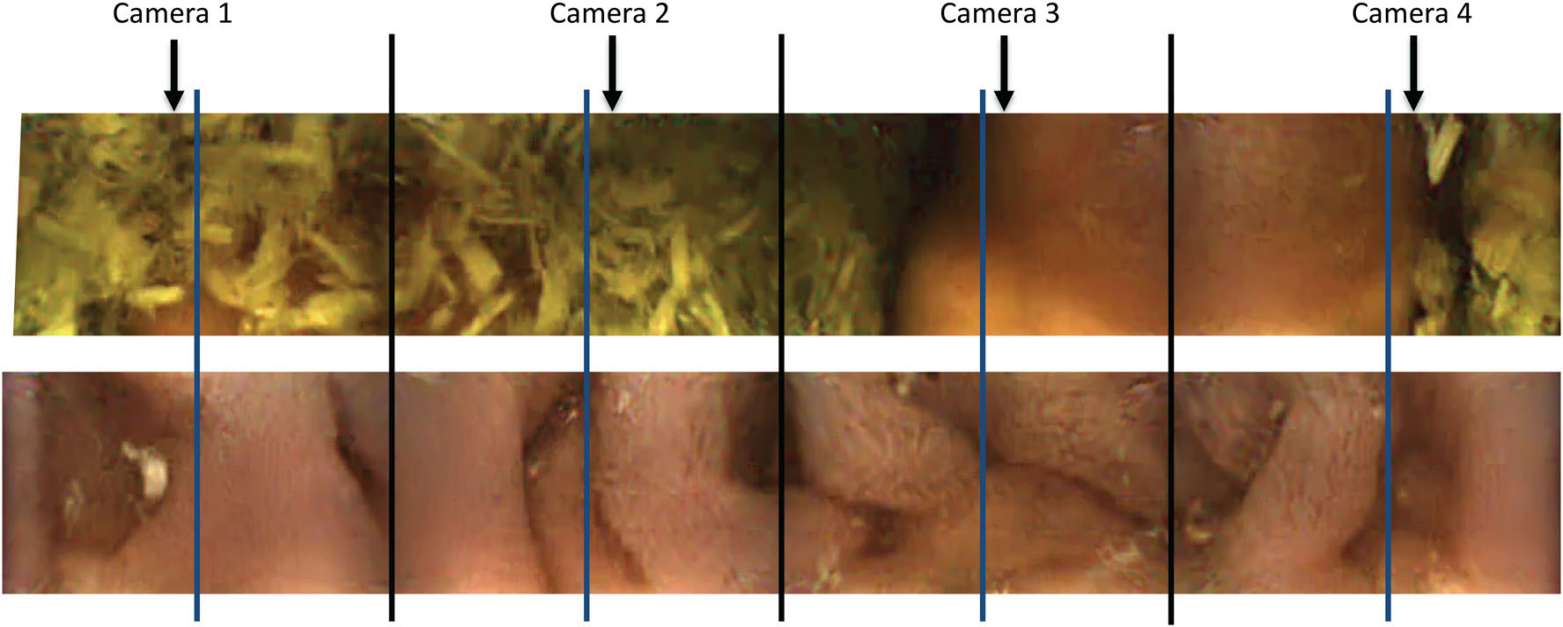
Two example full 360° frames obtained by the capsule (4 camera images, identified by the black lines). Each of the 4 images was divided into 2 sections (blue lines). If <50% was obstructed by feed material, that section was given a score of 1; all scores were then added together to give a total, objective score for the full frame (9‐point scale from 0 [fully obstructed by feed material] to 8 [no obstruction]). In the example frames, the top frame was scored 2 and the bottom frame was scored 8

Two observers, both board‐certified internists (R. L. and J. P.), evaluated the studies independently using a previously described visual analog scale^8^, ^15^ with modifications as follows (Figure 2): The proportion of visible mucosa in each full frame was scored on a 9‐point visual scale from 0 (fully obstructed by feed material) to 8 (no obstruction).^8^, ^15^ Each image captured by an individual camera was divided into 2 sections. If less than half of that section was obstructed by feed material, that section was given a score of 1, and all scores were then added together to give a total score for the full frame (Figure 2). An average of all full frame scores was taken for each section of the gastrointestinal tract. Amount of visible mucosa was reported as a percentage.

### 2.8 Clinical analysis of capsule images

An examination was also performed (J. P.) using the imaging software described above on a frame by frame basis of all the recorded frames for clinical interpretation and identification of lesions. The lesions were reported as descriptive interpretations based on clinical experience.

### 2.9 Histological interpretation

One horse (horse 5) used in the research trial was euthanized after the third protocol for reasons unrelated to the study. A postmortem examination was completed including thorough examination of several areas of the gastrointestinal tract with gross visible lesions for histopathological interpretation.

### 2.10 Statistical analysis

The data for transit times and battery life were reported as median (±IQR), and the data for image quality were reported as mean (±SD). Interobserver agreement was tested using the Pearson correlation coefficient using Statistix9 software (Analytical Software, Tallahassee, Florida).

## 3 RESULTS

### 3.1 Transit times

**FIGURE 3:**
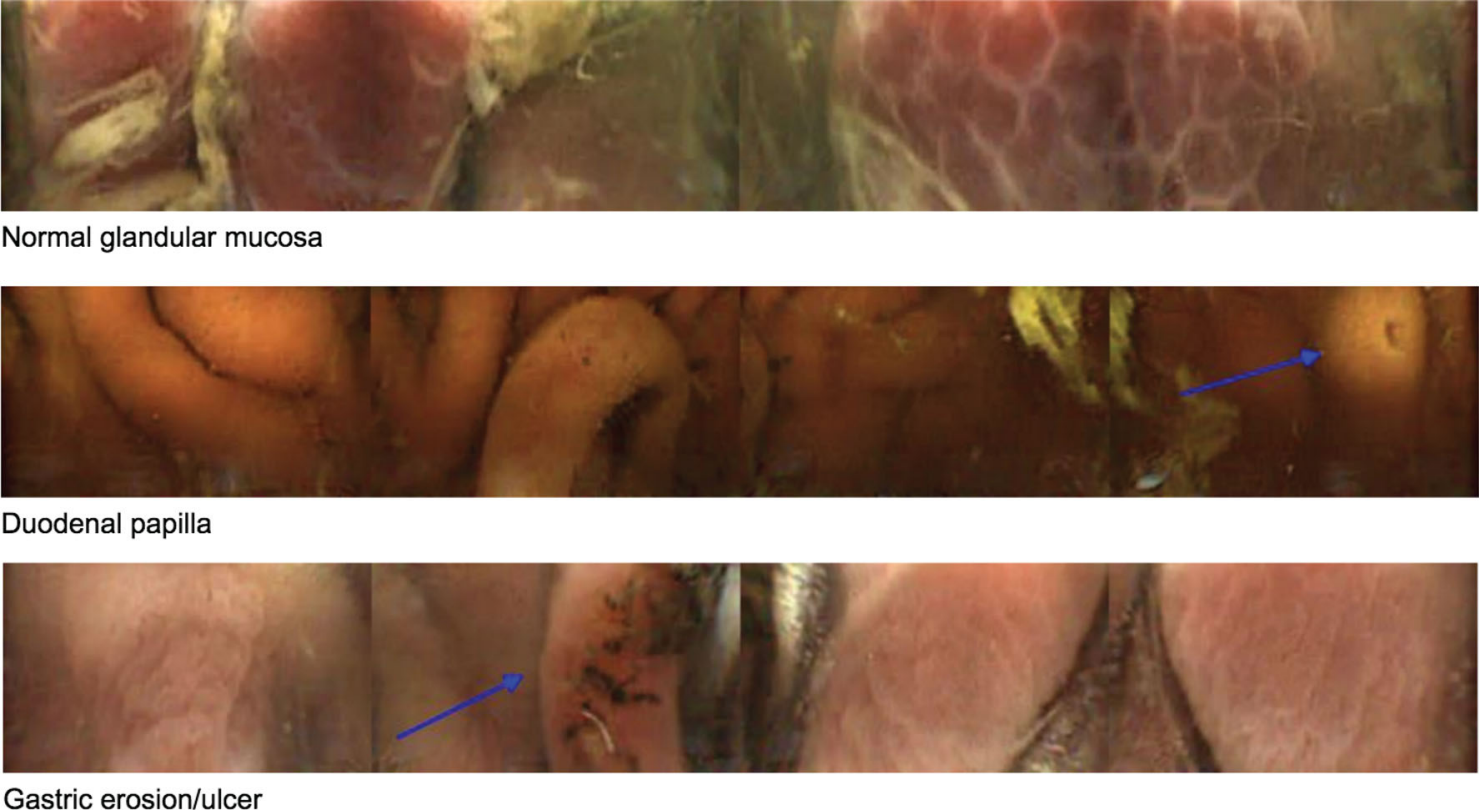
Stomach and pyloric view obtained by the capsule (3 frames). Top frame shows a normal glandular mucosa. Middle frame shows the duodenal papilla. Bottom frame shows ulcerations of glandular gastric mucosa

**FIGURE 4:**
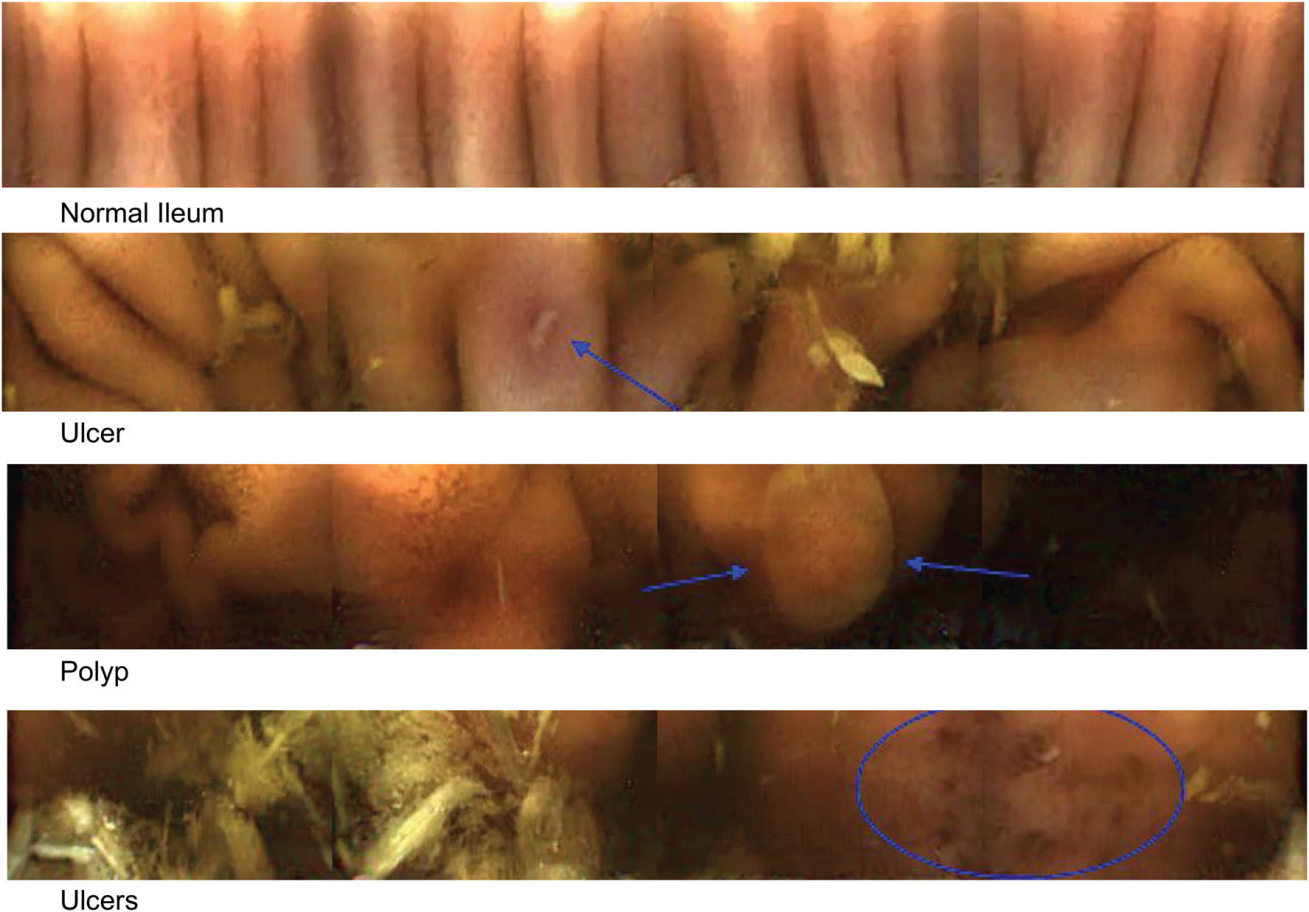
Small intestinal mucosa view obtained by the capsule (4 frames). Frames show normal ileal mucosa, ulcerations, and a small polyp

Transit times of the capsule through the gastrointestinal tract, total imaging time, total number of images taken per horse, and time to excretion for all 3 protocols are shown in Figure 1. Total imaging time was the amount of time that the capsule could capture images until either the onboard memory chip was full, or the battery‐life expired. The gastric mucosa had a very distinct appearance from the small intestinal mucosa (Figure 3 and 4). Passage into the cecum was also easily identified by the loss of visualization of the ileal mucosa (likely because of the large diameter of the cecum) and by the visualization of the ileocecal valve in some cases. The capsule entered the cecum during functional imaging life for 3 horses in protocols 1 and 3, and for 1 horse in protocol 2. No recordings of the colon could be obtained. The capsule was not recovered within the study period in 3 trials, with 2 capsules not being recovered from the same horse (Figure 1).

### 3.2 Image quality

In all protocols, the greatest percentage of visible mucosa was in the small intestine, with the greatest percentage of visible gastric mucosa being observed in protocol 1.

Visualization of the cecal mucosa was limited in all protocols. Mean percentages of visible mucosa in the stomach, small intestine, and cecum for each protocol are reported in Table 1. There was excellent interobserver agreement for scoring the mucosa visualization, with a Pearson correlation coefficient of 0.998.

**TABLE 1:** Mean percentage (%) ± SD of mucosa visualized by the endoscopy capsule based on a random selection of 30 image frames from the stomach and cecum, and 60 image frames from the small intestine, assessed on a 9‐point scale (n = 5 horses)

	Stomach	Small intestine	Cecum
Protocol 1	34 ± 27.3	38.1 ± 24.4	0.68 ± 0.26
Protocol 2	2.1 ± 2.1	23.96 ± 23.97	1.25^a^
Protocol 3	2.2 ± 1.4	25.4 ± 15.4	2.8 ± 4.5

^a^ One capsule recovered.

### 3.3 Interpretation of capsule images

**FIGURE 5:**
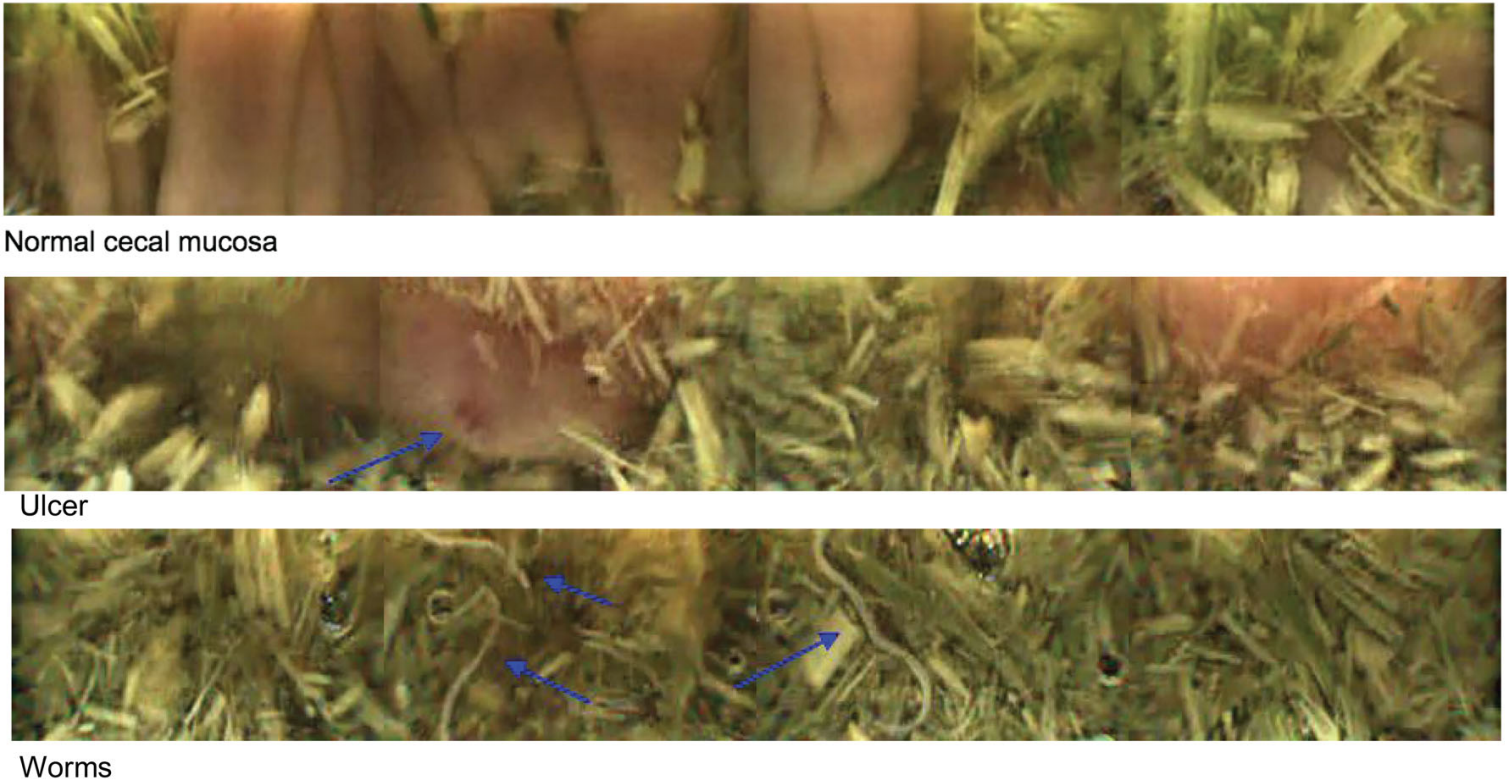
Cecal view obtained by the capsule (3 frames). Frames show normal cecal mucosa, an ulcer, and round worms

The capsule enabled visualization of normal gastric mucosa including both glandular and nonglandular areas, the pyloric antrum, and the pyloric‐duodenal junction (Figure 3). Gastric pathology visualized included areas of thickened and irregular mucosa and areas of erosion and ulceration (Figure 3). In the small intestine, the capsule enabled visualization of normal anatomical structures including the duodenal papilla and individual villi, as well as pathological areas of erosion, ulceration, and pinpoint hemorrhages (Figure 4). In the cecum the ileocecal junction, normal cecal mucosa and parasites were observed (Figure 5).

### 3.4 Histopathology results

Horse 5 was euthanized after the study. The lesions reported during the clinical examination of the video frames included thickened gastric mucosa and possible erosions and hematomas of the small intestinal mucosa. Gross lesions submitted for histopathology include edematous and irregular gastric mucosa and possible erosions and hematomas in the small intestine. Histopathological findings showed areas of mild, acute to subacute ulceration, and erosion in the nonglandular stomach with mild congestion of proprial and submucosal vasculature. In the small intestine, there was mild, multifocal, submucosal vascular congestion and ectasia, and several areas of markedly congested vessels expanding the submucosa, with mild dilatation of submucosal lymphatics.

## 4 DISCUSSION

Our study investigated the feasibility of using a wireless endoscopy capsule to visualize the gastrointestinal mucosa in horses, and assessed the effect of changes in preadministration preparation on both the transit time and the amount of gastrointestinal mucosa visualized. Using 3 different preadministration protocols, we showed that this endoscopy capsule provides a safe, practical, and relatively noninvasive method to visualize the mucosa of the stomach, the proximal and distal small intestine, and in some cases the cecum of the horse. Protocol 1, where horses had withheld feed for 24 hours and had 12 hours' water restriction before capsule administration, provided the highest percentage of visualization of the gastrointestinal mucosa. Furthermore, the capsule was recovered in all horses undergoing protocol 1. Unexpectedly, the addition of hand walks before administration of the capsule, and an increase in feed withholding time from 24 to 48 hours decreased both the percentage of visualization of the gastrointestinal mucosa, and the recovery of the capsule from the horses.

Strengths of our investigation include the crossover study design, where we assessed the effect of 3 preadministration protocols on the same clinically healthy horses. A weakness of the study design was that sedatives (xylazine and acepromazine) were used to facilitate nasogastric tubing for capsule administration. Both drugs have been shown to have a negative effect on gastrointestinal motility, although to a lesser degree than other commonly used sedatives.^16^, ^17^, ^18^ In practice, sedation might be required to deposit the capsule via nasogastric intubation. Importantly, in our study, xylazine and acepromazine administration did not increase gastric transit time or small intestinal transit time of the capsule. Several horses had the capsule administered without sedation for the first or second protocol or both, and required sedation for the second or third protocol or both.

There are strengths and limitations of both capsule endoscopy systems involving real time image transmission (eg, PillCam) as well as systems where the image is recorded, with image visualization after capsule retrieval (eg, ALICAM). An advantage of the endoscopy capsule used in our study compared to previously investigated capsules^8^, ^12^ was the longer battery life, probably aided by the “power‐saving mode” when the capsule is not moving. This maximizes battery life and total image acquisition time, which allowed for examination of the entire small intestinal tract, and in some cases of the cecum. However, the time for the capsule to exit the stomach is variable and has previously been identified as a limitation in real‐time wired endoscopy capsule use in horses.^9^ While the longer total battery life and “power‐saving mode” of the capsule used in our study bestows greater opportunity for diagnostic image acquisition aboral to the stomach than other capsule systems, it is still a limiting factor of this technology. Compared to the gastroscopy technique, that provides directional control of the endoscopic camera in real‐time, the capsule techniques will provide a more limited visualization of the squamous mucosa of the stomach. However, the capsule technique used in our study provided a good visualization of the pyloric area of the stomach, which can be more challenging to reach with the gastroscopy technique. Additionally, lesion location within a gastrointestinal segment is challenging with all types of capsule systems, because of an inability to accurately locate the device, as well as intermissions between image transmission or recording. However, while lesion *identification* is obviously of merit, lesion *localization* beyond the identification of a specific segment of the gastrointestinal tract is unlikely to change treatment recommendations for equine practitioners.

Normal anatomical features, such as the glandular and nonglandular portions of the stomach, the pylorus, the major duodenal papilla, individual villi in the small intestine, and the ileocecal junction, were identified. Images acquired had enough resolution to determine mucosal shape and color throughout the gastrointestinal tract up to and including the cecum. Interestingly, in these clinically healthy horses, the capsule also obtained images of thickened mucosa and areas of possible erosion or ulceration in the stomach and small intestine (see figures). Furthermore, lesions observed in capsule images obtained from the horse that was euthanized were consistent with both gross lesions observed during postmortem examination and histopathological findings from corresponding sections of the gastrointestinal tract. While some lesions were detected, further studies to characterize the types of lesions the capsule can reliably visualize in various disease states are warranted. Such sensitivity and specificity studies were outside the scope of our study and would require a postmortem examination on many clinical cases, which might be challenging. Last, the clinical relevance of the observed lesions has yet to be determined.

As expected, obstruction by feed material was a major limitation to image acquisition, which concurred with previous reports of other endoscopy capsule systems tested in horses.^8^, ^12^ Adequate preadministration preparation of the intestinal tract helps to mitigate this concern; 24 hours of feed withholding flushing with magnesium sulfate has previously been shown to be effective at improving visibility of the gastrointestinal mucosa.^8^ We did not use a magnesium laxative solution in our study to avoid potential adverse effects. In agreement with this previous report, we found that protocol 1, where horses had withheld feed for 24 hours before capsule administration, provided the highest percentage of visualization of the gastrointestinal mucosa. However, when the feed withholding period was increased to 48 hours, and the gastrointestinal tract was flushed with an electrolyte solution to stimulate motility of the small intestine and clear debris^19^, ^20^ (protocol 3), a marked decrease in the percentage of visible mucosa was observed.

While the capsule studied does not experience failure of image acquisition caused by poor connection with external sensors, as has been observed with other capsules,^8^, ^10^, ^11^, ^12^ a major limitation is that recovery is required for it to be used as a diagnostic tool. In our study, capsule excretion time varied widely between horses and between protocols; the different protocols did not appear to influence overall excretion time. As a diagnostic tool in a clinical setting, variability in the capsule excretion time might be problematic. Gastric emptying in horses can be prolonged by ulceration, ileus, impaction, pyloric strictures, and equine dysautonomia,^10^, ^18^ any of which could lead to a delayed capsule excretion time. While the horses enrolled in our study were all clinically healthy, 3 capsules were unrecovered after 24 days (including 2 from the same horse), potentially because of capsule retention or loss. Therefore, future studies on horses with gastrointestinal disease investigating risk factors for delayed excretion or capsule retention are recommended. Prokinetic drugs have been used in horses to promote motility in the event of ileus and investigation is warranted to assess if they might be useful in cases where the capsule appears to be retained.^21^, ^22^

The investigation of the feasibility of capsule endoscopy for use in foals is also warranted. It is possible that real‐time wired endoscopy capsules have greater utility in foals than in adult horses, because of the smaller body mass potentially resulting in less gaps in image transmission. The comparative advantages and limitations of different types of endoscopy capsule systems in foals are presently unknown. The progress of the capsule as it travels through the gastrointestinal tract of foals might be readily tracked using radiographic techniques and could give insight to common areas of retention.

Hand‐walking is a technique that is commonly utilized to stimulate motility after a small intestinal resection or laparotomy; however, there is a lack of evidence that exercise is directly linked to motility.^23^, ^24^, ^25^ In our study, increasing the amount of hand‐walking (protocols 2 and 3) did not influence capsule excretion time. Interestingly, the 2 horses that were housed in outdoor pens during protocol 3 passed their capsules very quickly (horse 1: 2 days; horse 2: 3 days), compared to their capsule excretion time during the previous 2 protocols (horse 1: 9 days and uncollected; horse 2: 10 and 7 days). It is plausible that these horses had increased ambulation during protocol 3 due to the increased area of their pens.

Feed withholding horses for 24 hours before capsule administration provided the highest percentage of visualization of the gastrointestinal mucosa; further refinement of preadministration protocols might help to increase the amount of unobstructed mucosa observed. This capsule was able to visualize regions of the small intestine to a greater extent than other endoscopic capsules. The use of this novel endoscopic capsule appears to be safe, practical, and relatively noninvasive in horses, and could offer valuable diagnostic information that would otherwise be unavailable to the practitioner.

## CONFLICT OF INTEREST DECLARATION

Dr J. Pomrantz was an employee of Infiniti Medical, LLC at the time the study was conducted. Dr. R. Léguillette did ad hoc consultations for Infinity Medical, LLC after the study was conducted. No other conflict of interest to declare.

## OFF‐LABEL ANTIMICROBIAL DECLARATION

Authors declare no off‐label use of antimicrobials.

## INSTITUTIONAL ANIMAL CARE AND USE COMMITTEE (IACUC) OR OTHER APPROVAL DECLARATION

This study was conducted in strict accordance with the recommendations of the Canadian Council of Animal Care. The research protocol was reviewed and approved by the University of Calgary Veterinary Sciences Animal Care Committee (AC15‐0152).

## HUMAN ETHICS APPROVAL DECLARATION

Authors declare human ethics approval was not needed for this study.
